# Structured data entry for narrative data in a broad specialty: patient history and physical examination in pediatrics

**DOI:** 10.1186/1472-6947-6-29

**Published:** 2006-07-13

**Authors:** Sacha E Bleeker, Gerarda Derksen-Lubsen, Astrid M van Ginneken, Johan van der Lei, Henriëtte A Moll

**Affiliations:** 1 Department of Pediatrics, Erasmus MC – Sophia, Rotterdam, The Netherlands; 2 Institute of Medical Informatics, Erasmus MC, Rotterdam, The Netherlands; 3 Emergency Department, Juliana Children's Hospital, The Hague, The Netherlands

## Abstract

**Background:**

Whereas an electronic medical record (EMR) system can partly address the limitations, of paper-based documentation, such as fragmentation of patient data, physical paper records missing and poor legibility, structured data entry (SDE, i.e. data entry based on selection of predefined medical concepts) is essential for uniformity of data, easier reporting, decision support, quality assessment, and patient-oriented clinical research. The aim of this project was to explore whether a previously developed generic (i.e. content independent) SDE application to support the structured documentation of narrative data (called OpenSDE) can be used to model data obtained at history taking and physical examination of a broad specialty.

**Methods:**

OpenSDE was customized for the broad domain of general pediatrics: medical concepts and its descriptors from history taking and physical examination were modeled into a tree structure.

**Results:**

An EMR system allowing structured recording (OpenSDE) of pediatric narrative data was developed. Patient history is described by 20 main concepts and physical examination by 11. In total, the thesaurus consists of about 1800 items, used in 8648 nodes in the tree with a maximum depth of 9 levels. Patient history contained 6312 nodes, and physical examination 2336. User-defined entry forms can be composed according to individual needs, without affecting the underlying data representation. The content of the tree can be adjusted easily and sharing records among different disciplines is possible. Data that are relevant in more than one context can be accessed from multiple branches of the tree without duplication or ambiguity of data entry via "shortcuts".

**Conclusion:**

An expandable EMR system with structured data entry (OpenSDE) for pediatrics was developed, allowing structured documentation of patient history and physical examination. For further evaluation in other environments, the tree structure for general pediatrics is available at the Erasmus MC Web site (in Dutch, translation into English in progress) [1]. The generic OpenSDE application is available at the OpenSDE Web site [2].

## Background

Use of the traditional paper chart as medium for recording and collecting patient data has limitations. Fragmentation of patient data due to use of scattered sources may interfere with the continuity of care [3]. In addition, research on non-standardized data in handwritten paper documents with poor legibility is labor-intensive and hampered by incompleteness [3]. Electronic medical record (EMR) systems are developed to address some of the limitations of paper-based recording [4-6]. The potential benefits of an EMR are many, both for patient care, quality assurance, research, and policy [4-11]. The main benefit of using an EMR is yielding data, all gathered in the same patient record. The ability to access the EMR at multiple locations overcomes the problem of physical paper records missing. Electronic data interchange (e.g. for transferring patients) is possible. Typed data benefit the legibility. In addition, the potential benefits of structured data entry (SDE), i.e. data entry based on selection of predefined medical concepts, in particular are uniformity of data, easier reporting of data (e.g. by a standard letter to the general practitioner), and more advanced decision support (e.g. by embedding clinical guidelines in the EMR), quality assessment, and patient-oriented clinical research. In the end, all these aspects together could result in a better and more complete documentation of patient data and a more efficient and better patient care [4,6,8,10-12].

However, structured recording of the medical narrative (i.e. patient history and physical examination) has proven to be a significant challenge [4,5,13]. Medical narrative data are diverse, and vary per discipline, per patient, and over time. Although broad specialties, for example internal medicine or pediatrics, acknowledge the importance of SDE [6,14], efforts to introduce EMR systems that include SDE for medical narrative have been limited to concise subject areas such as radiology or endoscopy [15-17]. Such applications for medical narrative of broad specialties has not been described in current literature.

Researchers are developing software environments that enable SDE of medical narrative; OpenSDE is one of those environments [18-20]. Broad specialties like pediatrics or internal medicine can be viewed as the ultimate test for SDE applications; the application will be challenged to allow recording of a wide range of narrative varying from straightforward to very complex problems covering all aspects of both patient history and physical examination. In addition, the application has to be embedded in daily practice and has to meet the specific needs of the clinician and require minimal extra work from the clinician [5,7].

In this paper, we report on the application of OpenSDE to general pediatrics. To support the structured documentation of narrative data of the broad domain of pediatrics, we customized the OpenSDE application for data obtained at pediatric history taking and physical examination. In addition, in line with the open source approach advocated by OpenSDE, we provide the resulting record to the user community for further evaluation and possible adoption in their own environment.

## Methods

### Materials

OpenSDE has been developed at the Erasmus Medical Center, department of Medical Informatics, to support the structured recording of patient data in any medical domain [20,21] and was used for the support of SDE pediatric narrative data, as it appeared to meet our requirements: flexibility in content, uniformity of the underlying structure, and provision of unambiguous and structured data. OpenSDE is able to accommodate data from a broad specialty in which the possibility of a detailed description of history taking and physical examination is necessary, and specific needs of each (sub)specialty can be customized. It provides interchangeability of data among disciplines (shared records), and changes in the content covered by SDE does not require any change in database structure or software [18-20]. OpenSDE presents data with explicit ordering, clear headings and subheadings, and custom views on data [22,23].

In OpenSDE, there is a thesaurus of medical terms. Since medical descriptions are by nature hierarchical, the medical terms are ordered as nodes in a tree structure, such that the path from the root to a node represents a medical concept in its context. The branches of each node represent its descriptors. Terms in the thesaurus may apply to more than one context: the item severity, for example, appears once in the thesaurus of terms, but many times in the tree at different locations, e.g. severity of dyspnea and severity of abdominal pain. These 'severity' nodes represent different medical concepts. In case a medical concept (hence with the same meaning, e.g. vomiting) applies in more than one context, one node has the descriptors, while other nodes, representing that same concept, have a reference (called "shortcut") to the node with the descriptors. This tree structure as a whole defines which medical concepts in what combinations can be used to create meaningful medical descriptions.

**Figure 1:**
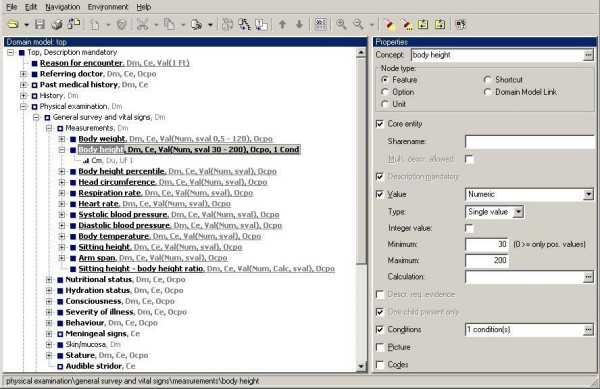
Editor for creating the tree structure. The right side of the screen shows the properties associated with the selected node 'body height'. 'Minimum' and 'Maximum' indicate the plausible range for body height. A condition is added to accommodate rare, but potentially valid values. The 'Codes' field can be used to assign identifiers from one or more standard terminologies. Each node is displayed with its associated term from the thesaurus. Unique numeric identifiers are meaningless and hidden. These can be shown on demand.

The tree does not only represent the ordering of the medical descriptors: each node in the tree has properties that define the data type of the node and the constraints for data entry (figure 1).

### Methods

This study was approved by The Institutional Review Board of the Erasmus MC in Rotterdam, The Netherlands. For the content of the EMR, medical concepts and its descriptors in pediatric history taking and physical examination were reviewed in close consultation with two pediatricians (GDL, HAM), using the national standardized pediatric medical record (comprising the sections: problem list, the general history tract, specific history tracts, the physical examination, course, and neonatology) [24] and a standard pediatric textbook [25]. Subsequently, this content was modeled into an OpenSDE tree structure. For the construction of the tree structure a standard terminology was not used as such terminologies limit freedom of expression [26]. It is, however, possible to associate nodes in the tree with concepts in a standard terminology to promote consistent mapping of entered data [27]. Reference values for continuous values (e.g. vital signs) were defined according to the textbook [25].

To establish how comprehensive, easy and valid the thesaurus and tree structure was five volunteer (trainee) pediatricians performed an initial assessment with OpenSDE in a research setting. Each (trainee) pediatrician was asked to select at least five medical assessments of patients, who had visited the ambulatory care with a new presenting problem. The pediatricians reported to the development team any difficulties with navigating the tree, incompleteness or inconsistency they encountered. Subsequently, the concepts were arranged in a more logic (intuitive) way and the tree structure was adapted.

## Results

### Structure of the tree

**Figure 2:**
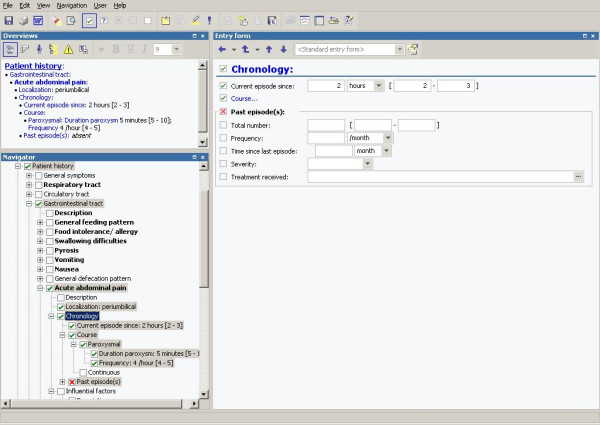
EMR system with structured data entry for pediatric patient history and physical examination data with a view on the standard entry form on the right side.

As part of an EMR system, we constructed a SDE tree for pediatric patient history and physical examination data (figure 2). The top of the tree in this EMR system starts with history and physical examination. History divides in branches with the specified concepts past medical history (including immunization status), family history, allergies, social history, current medications and the current chief complaint. The chief complaint exists of a general tract and 14 specific history tracts (the respiratory-, circulatory-, gastrointestinal-, and urogenital tract, ear-nose-throat, skin, organs of sense, the nervous-, endocrine-, locomotor- and hematological system, feeding history, prenatal and delivery history, growth and developmental history). Each of these, in total 20, history concepts splits again in 5 to 25 sub branches. For instance, the gastrointestinal tract subdivides in 10 main concepts: general feeding pattern (including undernutrition, overnutrition, and appetite, which is linked to feeding history from the list of specific history tracts), intolerance- or allergy for food, swallowing difficulties, vomiting, nausea, general defecation pattern (including diarrhea, constipation, mucous stools, painful defecation and bloody stools), abdominal pain (acute and chronic), pyrosis, flatulence and fecal incontinence. Sub branches are in the end described by 4 to 15 general attributes, like duration, severity, timing and setting of complaint, influencing factors of complaint and associated manifestations.

Physical examination splits in 11 branches (general survey and vital signs, head and neck, thorax, abdomen, skin, lymph nodes, limbs, genitalia, anus and rectum, nervous system, and spine), of which each forks as well. In total, the thesaurus consists of about 1800 items, used in 8648 nodes in the tree with a maximum depth of 9 levels. Patient history contained 6312 nodes, and physical examination 2336.

### Data entering

**Figure 3:**
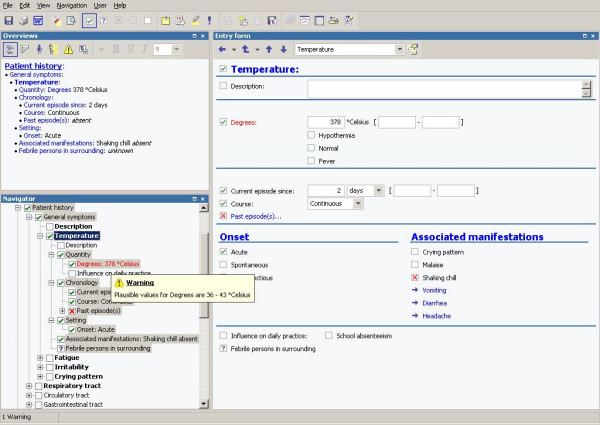
Continuous data validation: a warning appears entering an implausible value.

By selecting a concept (e.g. acute abdominal pain) in the tree, displayed on the left of the screen, a data entry form is displayed on the right with the descriptors (e.g. localization, timing, course) of the selected concept as options for data entry (figure 2). Data entry is accomplished primarily with the mouse: the user selects from pull-down boxes and pick-lists. Concepts can be marked as "present", "absent" or "unknown", and no default was set. Free text annotations can be added to any finding or description. Values, e.g. body temperature, have to be entered using the keyboard. If an implausible value is entered for a particular item, e.g. 378 degrees Celsius for body temperature, a warning appears and the item will be presented in red (figure 3). Clinicians are not forced by the system to change the data.

A search option is available to locate a specific item in the tree. Entering a search term results in a number of hits, including synonyms. Each hit is displayed with its pathway, showing its position in the tree.

To facilitate data entry, we defined several (226) "shortcuts" (reference from one node to another), by which searching and scrolling up and down the tree, as well as repeatedly recording of data in multiple places, are avoided. For example, fever is described in the general history tract. As fever is an important associated manifestation of many symptoms, it has to be addressed in several history tracts as well (e.g. in the history taking of coughing, which is described in the respiratory tract, or in the history taking of vomiting, which is described in the gastrointestinal tract). When the clinician selects, for example, the "shortcut" 'fever' in the respiratory tract, the program directly jumps to the descriptors of fever in the general history tract, where the data on fever will be stored. After completing data entry on fever in the general tract history, it is possible to return to the previous position in the tree (e.g. coughing in the respiratory tract) immediately.

**Figure 4:**
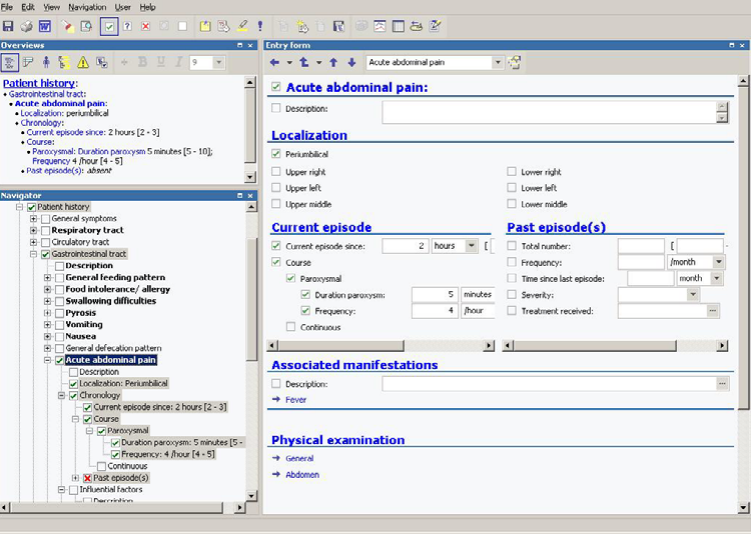
EMR system with structured data entry for pediatric patient history and physical examination data with a custom entry form on the right side.

At each moment the clinician is free to choose the starting point and endpoint (degree of detail) of data entry. Moreover, the user can, without affecting the underlying data representation, define an unlimited number of custom entry forms that contain a selection of nodes in the tree for a specific medical problem or disease entity. On such a custom form, medical concepts from different positions in the tree can be combined. Figure 4 shows a custom form for acute abdominal pain that allows the physician to record the signs and symptoms, including localization and timing of the complaint, and physical examination of the abdomen.

The program provides the possibility to export entered data to a text editor or to Microsoft Word. The exported data will comprise basic administrative patient data, the date of data collection, the name of the clinician who recorded the data and the collected data from history taking and physical examination in outline (indented) display. The text can be edited and completed by for instance a description of the interpretation of the presented clinical problem, a diagnosis or a treatment strategy. The resulting report can be used as a letter to the general practitioner.

## Discussion

This paper explores whether a previously developed SDE application (called OpenSDE) can accommodate the structured recording of medical narrative data of a broad specialty. An expandable EMR system with OpenSDE for general pediatrics is developed, allowing structured documentation of patient history and physical examination. The resulting thesaurus contains 1800 terms, and the OpenSDE tree contains 8648 nodes (patient history and physical examination contain 6312 and 2336 nodes, respectively).

Developing interface terminologies poses a range of scientific issues that have to be, implicitly or explicitly, addressed [13]. The ultimate test of our approach will be the use of the system in daily clinical care. During the development of the system, coverage and user friendliness were assessed by physicians entering actual cases; this, however, must be viewed as an initial assessment only.

The content and structure of data in the EMR system may change over time, for example, for expansion of the tree. Previously stored data, however, must remain fully accessible. OpenSDE provides this functionality and thus provides a flexibility that is very important for acceptability of an EMR system among future users.

The main lesson learned from the development of an EMR system with SDE for a broad specialty concerns navigating the tree. To meet this difficulty, the user has the option to use customized entry forms without affecting the underlying data representation. When a medical concept is relevant in more than one context, the user is redirected via "shortcuts" to the one unique node where this concept can be described. Duplicate or inconsistent description of one concept is thus avoided. The "shortcuts" further allow return of the user to his/her last position in the tree structure. Nonetheless, the use of custom forms and "shortcuts" probably will not be conclusive and navigation remains a challenging subject for future developers. This project taught us furthermore that a balance has to be found between research objectives, and demands for patient care. The modeling of free text annotations in the tree structure, enabling the author to describe in his own words a concept or a finding, is an example of a (partial) concession made to the structured entry of data. Thirdly, we experienced that, although pediatric expertise was available during construction of the tree, medical assessments by clinicians who were 'new users', led to adaptations of the tree.

Researchers have developed a number of electronic patient record systems. In the Netherlands, for example, almost all general practitioners use a computer to record patient data [7,9]. Typically, these records provide some overall structure and include some coding (e.g., the International Classification for Primary Care, the ICPC). Data dealing with patient history and physical examination, however, are typically recorded as free text. Especially for general and broad specialties, such as pediatrics and internal medicine, in which detailed description of history taking and physical examination is needed, an EMR system with SDE for data from history taking and physical examination has, to our knowledge, never been realized.

In this paper, a pediatric context is used to explore whether the OpenSDE application can be used to model narrative data of such a broad specialty. At present, OpenSDE is also being applied to several other medical domains [28]. An additional benefit of the developed applications is the possibility that different specialists can share their records, while working with the same recognizable user interface. A common user interface across different specialties enhances familiarity with the system and contributes to the ultimate use of the EMR system in practice.

Before widespread implementation of an EMR, the system should be rigorously evaluated and security and ethical aspects must be addressed [8,29,30]. In a separate study, we evaluated our pediatric EMR system with respect to its completeness, uniformity of reporting, and its usability in clinical practice. The physicians share a positive attitude towards the SDE application and the EMR system appears to be a promising application for the support of physician data entry in general pediatrics [31].

Currently, OpenSDE functionality is being implemented in our Hospital Information System, following a pilot in the pediatric emergency department. Among future plans for applications in the pediatric EMR system with SDE are the embedding of age-based normal ranges of measurements for vital signs and other physiological parameters, clinical decision rules and graphic display of growth data and special calculations of growth patterns [32]. Further prospects are incorporation of reminder systems (e.g. for immunization or differential diagnosis [11], selection of patient subgroups (e.g. for prevention strategies) and support of adequate drug dosing.

## Conclusion

In conclusion, OpenSDE, a generic SDE application to support the structured documentation of narrative data, was tailored to the broad domain of general pediatrics. The resulting expandable pediatric EMR allows structured collection of pediatric history and physical examination data. For further evaluation in other environments, the tree structure for general pediatrics is available at the Erasmus MC Web site (in Dutch, translation to English in progress) [1]. The generic OpenSDE application is available at the OpenSDE Web site [2].

## Abbreviations

EMR: electronic medical record

SDE: structured data entry

## Competing interests

The author(s) declare that they have no competing interests.

## Authors' contributions

SEB modeled the tree structure and had primary responsibility for writing the manuscript.

GDL participated in the conception and design of the study, supervised the medical part of the modeling of the tree structure and contributed to drafting the manuscript.

AMvG participated in the conception and design of the study, supervised the technical part of the modeling of the tree structure and contributed to writing of the manuscript.

JvdL had primary responsibility for the conception and design of the study and supervised the writing of the manuscript.

HAM had primary responsibility for the conception and design of the study, supervised the medical part of the modeling of the tree structure and supervised the writing of the manuscript.

All authors read and approved the final manuscript.

## Pre-publication history

The pre-publication history for this paper can be accessed here:


